# A 2 kW, 8 GHz-Linewidth Yb-Doped Polarization-Maintained Fiber Laser with Quasi-Flat-Top Pseudo Random Binary Sequence Phase Modulation for SBS Suppression

**DOI:** 10.3390/nano13081329

**Published:** 2023-04-10

**Authors:** Dong Joon Kim, Joonhoi Koo, Seung Won Jun, Hwanseong Jeong, Hwihyeong Lee, Jung Hwan Lee, Minsik Jo

**Affiliations:** Ground Technology Research Institute, Agency for Defense Development, Daejeon 34186, Republic of Korea

**Keywords:** fiber laser, narrow linewidth, polarization-maintaining fiber, pseudo random binary sequence

## Abstract

We demonstrated a narrow-linewidth high-power Yb-doped polarization-maintaining (PM) fiber laser with near-diffraction-limited beam. The laser system consisted of a phase-modulated single-frequency seed source and four-stage amplifiers in the master oscillator power amplifier configuration. A quasi-flat-top pseudo random binary sequence (PRBS) phase-modulated single-frequency laser with a linewidth of 8 GHz was injected into the amplifiers for suppressing stimulated Brillouin scattering. The quasi-flat-top PRBS signal was readily generated from the conventional PRBS signal. The maximum output power was 2.01 kW with polarization extinction ratio (PER) of ~15 dB. The beam quality (M2) was less than 1.3 over the power scaling range.

## 1. Introduction

High-power fiber lasers with narrow linewidth are required in various regimes, such as nonlinear frequency conversion [1,2], gravitational wave detection [3], and beam combining [4,5,6]. Beam combining of fiber laser is one of the most effective methods to increase the output power while maintaining the benefits of the fiber laser. However, it puts high demands on beam quality, linear polarization, and linewidth of the laser sources and requires even higher power levels. Therefore, it is important to overcome the limitations, such as power scaling, nonlinear effects, and the stimulated Brillouin scattering (SBS) effect in particular, caused by the narrow linewidth of the laser source and transverse mode instability (TMI). In order to suppress the SBS effect, various approaches have been reported, such as applying a thermal gradient [7,8,9], designing a special fiber with acoustic layers [10,11,12], metal-coated fibers [13], and employing a phase-modulated laser source. Nowadays, there are several methods for obtaining a phase-modulated laser beam as a seed source, including sine-wave phase modulation [14,15], white noise signal (WNS) phase modulation [16,17,18,19], pseudo-random binary sequence (PRBS) phase modulation [20,21,22], and so on. In order to mitigate TMI effect, low-NA fibers [23], tight coiling [24], shifting the pump wavelength [25,26], pump modulation [27], and bi-directional pumping schemes [28,29] are among the approaches that have been proposed and adopted. In particular, the flat-top-shaped PRBS modulation technique, which is a great technique to suppress the SBS, has been recently reported [30,31,32]. A flat-top-shaped PRBS signal can be generated through an arbitrary signal generator and a modified PRBS can be obtained through field-programmable gate array (FPGA) programming. Recently, in order to suppress nonlinear effects, many high-power fiber amplifiers with narrow linewidths and diffraction-limited beams using the aforementioned techniques have been reported.

In general, kW class fiber amplifiers with linearly polarized output beams can be classified as non-polarization-maintained (PM) fiber amplifiers with polarization control systems and PM fiber amplifiers. Suppression of nonlinear effects in a high-power narrow-linewidth PM fiber amplifier is very challenging given that the effective Brillouin gain coefficient in PM fiber is typically higher as compared to the non-PM fiber [33]. Therefore, a non-PM fiber laser system is more advantageous than a PM fiber laser system for suppressing the nonlinear effects. However, a non-PM fiber laser system could be complicated due to the additional equipment required, such as a polarization controller and monitoring device to check the polarization states of the laser beam. In the last few years, many researchers made great progress in the power scaling of the narrow-linewidth fiber amplifiers using PM fibers. In 2015, a 560 W fiber amplifier with a linewidth of <5 GHz was reported, which used a phase-modulated seed source from an acoustic-optical driven source [34]. An 818 W PM fiber amplifier with a linewidth of <7 GHz was demonstrated based on the PRBS phase modulation in 2019 [22]. Moreover, a 1.5 kW PM fiber amplifier with a linewidth of 13 GHz was reported, based on WNS phase modulation, in 2019 [18]. A 3.25 kW PM fiber amplifier with a linewidth of 20 GHz was demonstrated based on the arbitrary waveform generator (AWG) phase modulation in 2021 [35].

In this study, we report the experimental demonstration of a 2.01 kW diffraction-limited beam-quality all-PM fiber amplifier with a slope efficiency of 77%, using the seed source modulated by a quasi-flat-top PRBS signal at 8 GHz, in a master oscillator power amplifier (MOPA) configuration. For enhanced TMI threshold, a bi-directional pumping configuration was adopted. The measured beam quality was 1.29 at the maximum output power of 2.01 kW, and a TMI effect was not observed. Upon comparison of the quasi-flat-top and conventional PRBS modulation, it was shown that the quasi-flat-top PRBS modulation could effectively suppress SBS.

## 2. Experimental Set-Up

**Figure 1:**
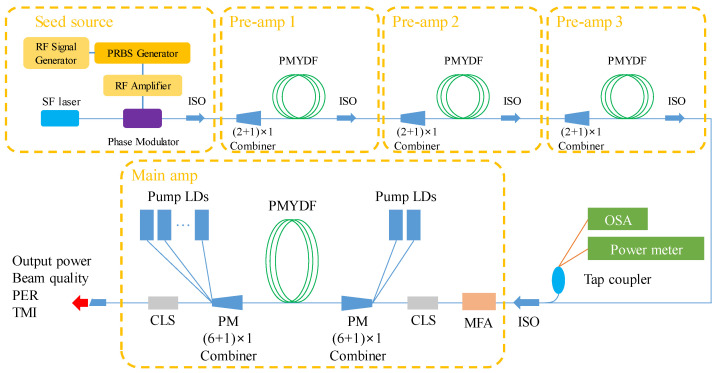
Experimental setup of the all-PM fiber amplifier. SF laser, single frequency laser; PMYDF, polarization-maintaining Yb-doped fiber; ISO, isolator; CLS, cladding light stripper; LD, laser diode.

We used a commercially available Yb-doped double-clad fiber (DCF) as a gain medium with a direct bi-directional pumping configuration in the high-power multi-stage MOPA system. The experimental setup of the high-power fiber laser with narrow linewidth is illustrated in Figure 1. To suppress the SBS, a phase-modulated single-frequency laser was used. For the phase modulation, PRBS signal, a phase modulation technique generally used for high-powered narrow-linewidth fiber lasers, was adopted. The PRBS signal was generated from a commercially available PRBS generator (PRBS45, Advantec, Tokyo, Japan) equipped with a radio frequency (RF) signal generator. The PRBS signal was then amplified through a RF amplifier. A flat-top-shaped PRBS signal, which is an efficient way to suppress the SBS, was generated. The single-frequency laser modulated by a phase modulator with ~8 GHz flat-top PRBS signal was injected into an amplifier consisting of a three-stage pre-amplifier and a main amplifier stage. The output power of the PRBS phase modulated seed source was measured to be 10 mW at the center wavelength of 1064.6 nm. The seed source was first amplified to be ~150 mW by adopting a single mode fiber first-stage pre-amplifier. The gain fiber used in the first-stage pre-amplifier was double-clad Yb-doped PM fiber with a core/clad diameter of 10/125 um, which was pumped by a 976 nm laser diode via a (2 + 1) × 1 pump/signal combiner. Due to the absorption coefficient of ~4.8 dB/m at 976 nm, the length of the Yb-doped fiber (YDF) used was ~3.0 m. The fiber length was chosen with respect to the nominal 13 dB absorption, which is required for adequate pump absorption. The amplified laser beam was injected into the second-stage pre-amplifier and the third-stage pre-amplifier, which had the same configuration as the first-stage pre-amplifier. The laser beam was boosted to ~2 W and ~30 W, respectively. The residual pump beam was stripped out by cladding light strippers (CLSs), which were inserted after the YDFs. High-power isolators were inserted after the CLSs to protect the fiber components by preventing backward light from the following stages. The main amplifier was based on a Yb-doped PM fiber with a core/clad diameter of 25/400 um as a gain medium, which was coiled with the minimum diameter of ~76 mm on an aluminous plate with the appropriate U-groove for increasing bending loss of the high-order mode to suppress the TMI effect. The cladding absorption coefficient was about ~1.8 dB/m at 976 nm and a fiber of ~7.3 m was used in amplifier. The YDF was bi-pumped by a 976 nm wavelength-stabilized laser diode via a (6 + 1) × 1 pump/signal combiner. The unabsorbed pump laser beam was stripped out by a high-power CLS. The output laser beam was collimated via a coreless fiber end-cap. The spectrum of the amplified laser beam was measured by an optical spectrum analyzer (AQ6370D, manufactured by Yokogawa, Tokyo, Japan). The beam quality (M2) was measured by a M2 m (Beam Squared, manufactured by Spiricon, North Logan, UT, USA). The output power and backward power were monitored by Ophir (5000W-LP2-50) and Thorlabs (PM100USB), respectively.

## 3. Experimental Results and Discussions

**Figure 2:**
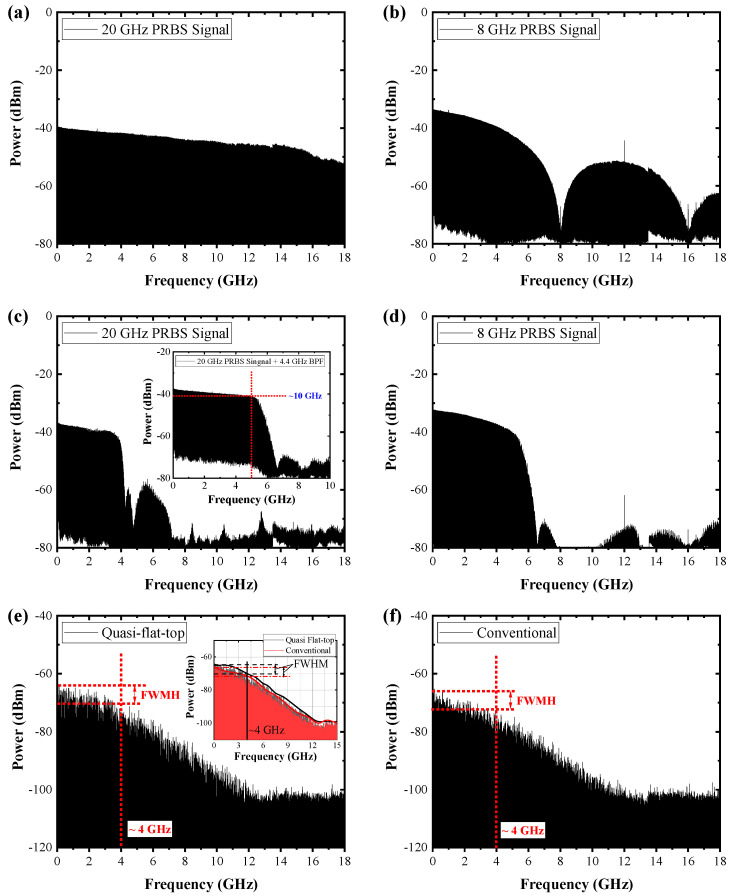
Measured RF spectra of the generated PRBS signal from PRBS generator: (**a**) at 20 GHz; (**b**) at 8 GHz. Measured RF spectra of filtered PRBS signal: (**c**) 3.4 GHz BPF (inset: measured RF spectrum with 4.4 GHz BPF); (**d**) 4.4 GHz BPF. Measured linewidth of the seed source with phase modulation: (**e**) 8 GHz quasi-flat-top PRBS (inset: multiplot graph with (**e**,**f**)); (**f**) 8 GHz-conventional PRBS modulation.

In order to increase the SBS threshold, a quasi-flat-top-shaped PRBS modulation with a band pass filter (BPF) was adopted in this work and the generated PRBS signals are shown in Figure 2. The target frequency of the PRBS modulation signal was 8 GHz. Thus, broader PRBS signals than 8 GHz in frequency were firstly generated from the PRBS generator to obtain a quasi-flat-top PRBS signal, as shown in Figure 2a. Such a broad 20 GHz signal was filtered by a 3.3 GHz BPF, as shown in Figure 2c. The linewidth was measured as ~4 GHz, which means 8 GHz linewidth. The inset figure of Figure 2c shows the filtered signal with a 4.4 GHz BPF. In this case, the linewidth is slightly larger than 8 GHz due to its stiffened edge near 4 GHz. For comparison with a quasi-flat-top PRBS modulation, we also measured a conventionally filtered PRBS signal, as shown in Figure 2b,d. The frequency of the applied PRBS signal was 8 GHz and then it was filtered by a 4.4 GHz BPF. The filtered signals were injected into a phase modulator within the seed source. The single-frequency laser was modulated by the phase modulator with those signals. The linewidths of the seed sources, measured by a self-heterodyne interferometer, are shown in Figure 2e,f, respectively. Given that the linewidth of the laser we used in our experiments was very narrow, it was difficult to accurately measure the laser linewidth using an optical spectrum analyzer. Thus, a self-heterodyne interferometer with the combination of both a photodiode and a RF spectrum analyzer was generally used to measure the linewidth of lasers, which has been used in previously reported works to measure the linewidth of the high-power fiber lasers [21]. The top of the measured linewidth of the seed source modulated by the 8 GHz quasi-flat-top PRBS signal is nearly flat-shaped, compared to that of the seed source modulated by the 8 GHz conventionally modulated PRBS signal. The inset within Figure 2e shows the multi plot of both linewidths, which shows that the black line, the quasi-flat-top PRBS-modulated seed source, has a nearly flat-top shape.

**Figure 3:**
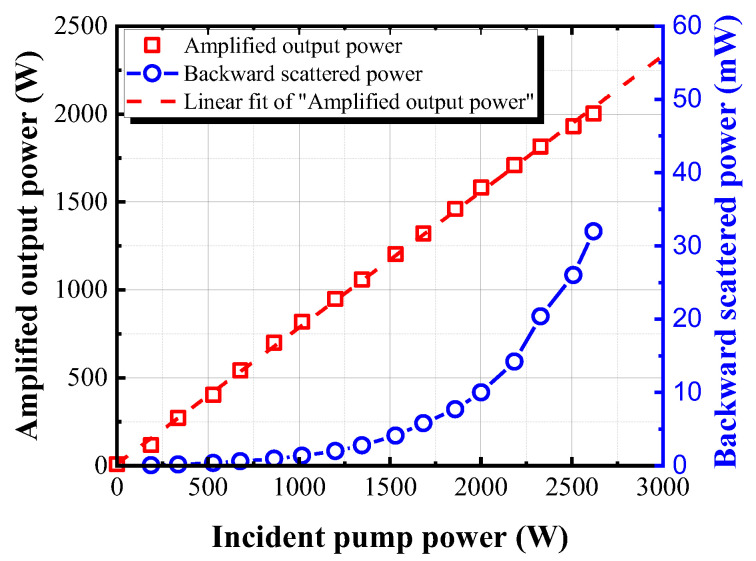
The measured amplified output power (red) and backward scattered power (blue) as a function of incident pump power.

To characterize the amplification of our fiber amplifier, we firstly measured the output power as a function of the incident pump power, as shown in Figure 3 (red curve). The achieved maximum output power was 2.01 kW with a slope efficiency of 77% without power roll-over. The blue curve in Figure 3 shows the backward scattered power. Experimentally it has been reported that backward reflectivity of 0.01~0.1% indicates the amplifier working around the SBS threshold [36,37]. Therefore, in our experiment, the backward reflectivity of the main amplifier reaching 0.01% was defined as the SBS threshold. As shown in Figure 3 (blue curve), the measured backward scattered power at the maximum output power in the main amplifier was about 32 mW. In other words, this means that the backward reflectivity was about 0.0013%, which was still lower than SBS threshold of 0.01%.

**Figure 4:**
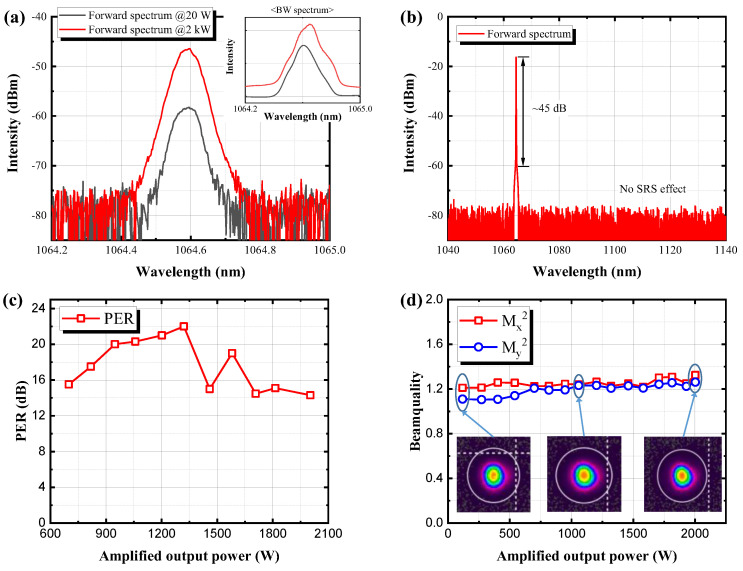
The measured output characteristics: (**a**) forward and backward (inset) beam spectrum; (**b**) forward output spectrum in a wide wavelength range at the maximum output power; (**c**) PER; (**d**) beam quality.

One of the advantages of the phase-modulated single-frequency seed source is that, unlike a general seed source, such as distributed feedback laser diodes (DFB-LD) and the output from a laser cavity without phase modulation, the linewidth of the amplified output beam is maintained regardless of the amplified output power due to suppression of new frequency generation of the laser, induced by self-phase modulation (SPM) [38,39]. Thus, the linewidth of the amplified output of the modulated single-frequency source is the same before the amplification process, as shown in Figure 4a. The inset figure is the backward spectrum for SBS monitoring at the maximum output power. The difference of the spectrum peak between Rayleigh scattering beam and SBS beam was about +5 dB. Figure 4b indicates the output beam spectrum in a wide wavelength range at the maximum output power. It shows that the stimulated Raman scattering (SRS) effect was not observed and the amplified spontaneous emission (ASE) was suppressed in a wide wavelength range. The optical to signal noise ratio was about 45 dB. Figure 4c shows the measured polarization extinction ratio (PER) at different output laser powers. As shown in Figure 4c, the measured PER changed between 14.6 dB and 22 dB. At maximum output power, the PER was about 15 dB. We also measured the beam quality during the power scaling process, as shown in Figure 4d. The inset in Figure 4d shows the measured beam profiles at different output powers. The measured beam quality values in the x direction and in the y direction were 1.324 and 1.261, respectively, thus indicating the amplifier operates at the nearly diffraction-limited beam.

**Figure 5:**
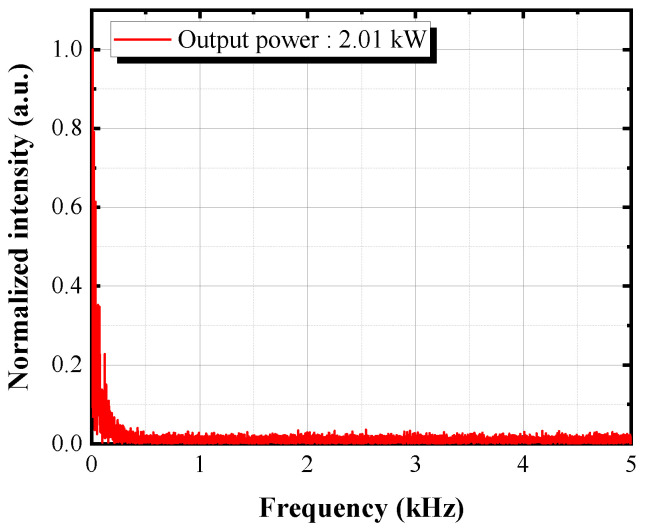
Power stability of the output beam at the maximum output power.

The temporal trace of the forward output beam was measured to monitor the TMI effect. Applying Fourier transform on the temporal traces to calculate the corresponding Fourier spectrum, we obtained the frequency distribution of the output beam stability in the range of 0~400 Hz at the maximum output power, as shown in Figure 5, which means that no TMI was found during the power scaling process in this amplifier [40,41].

**Figure 6:**
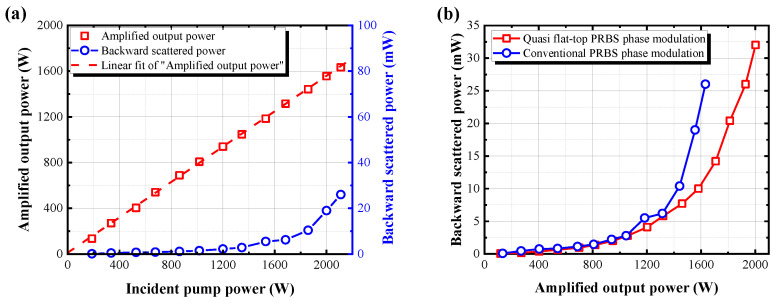
(**a**) The measured output power and backward scattered power as a function of the incident pump power based on the typically phase-modulated seed source; (**b**) The measured backward scattered power with different phase modulation conditions.

To prove the performance of the proposed PRBS technique, we also measured the output characteristics of the amplified seed laser using conventional PRBS phase modulation at 8 GHz, as shown in Figure 6. The PRBS modulation frequency of 8 GHz with a band pass filter of 4.4 GHz and *n* = 9 pattern allowed power scaling of the output to 1.63 kW. The applied electrical signal of PRBS signal is shown in Figure 2d. The amplified output power and backward scattered power were measured as a function of the pump power, as shown in Figure 6a. The output power linearly increased without power roll-over. As shown in Figure 6b, the SBS threshold for quasi-flat-top PRBS phase modulation is higher than that for conventional PRBS phase modulation. These results show that the proposed quasi-flat-top PRBS phase modulation could overcome the limitation of power scaling by nonlinear effects.

Finally, we present a comparison of the output beam properties of our PM fiber laser with those of previously reported narrow-linewidth PM fiber lasers. The results are summarized in Table 1. In terms of output power, the 3.25 kW fiber amplifier that incorporated an AWG phase-modulated seed source exhibited the best performance [35]. However, we found no reports in the literature of a high-power fiber amplifier with narrow linewidth less than 10 GHz. To the best of our knowledge, our PM fiber laser with a linewidth of 8 GHz is the first 2 kW PM fiber amplifier incorporating the quasi-flat-top PBBS phase-modulated seed source. As a matter of fact, many similar works have been already reported the narrow-linewidth high-power fiber lasers using flat-top modulation techniques [31,32,33]. Most of the previous reports used additional complicated coding, such as programmed AWG and FPGA. However, the proposed technique of this work can be used from the conventional modulation signal by filtering the broad conventional PRBS modulation signal, which does not need additional and complicated programing. Through the proposed technique of this work, a 2 kW laser with a linewidth of 8 GHz has been obtained. It is noted that such the output with the narrow linewidth is the highest output power, with linear polarization less than 10 GHz linewidth. Thus, we believe that this work is meaningful work.

## 4. Conclusions

We experimentally investigated a linearly polarized, 2.01 kW fiber laser based on a polarization-maintaining fiber amplifier with a linewidth of 8 GHz at 1064.6 nm. The PER and beam quality were measured to be 15 dB and 1.29, respectively, at the maximum output power of 2.01 kW. To suppress the SBS and TMI, both PRBS phase modulation and a bi-directional pumping configuration were used. In particular, quasi-flap-top PRBS phase modulation which can be readily generated from conventional PRBS signal was adopted. Through this technique, a relatively flat PRBS signal was readily generated and then used for phase modulation into the phase modulator to suppress SBS. The effectiveness of quasi-flat-top PRBS-modulated seed source was also shown by comparing the output power characteristics between the quasi-flat-top PRBS seed and the conventional PRBS-modulated one. The power scaling of the conventional PRBS-modulated one with same linewidth of 8 GHz was limited by 1.6 kW due to SBS. We believe our experimental results could be applicable for narrow-linewidth high-power fiber lasers.

## Figures and Tables

**Table 1:** Comparison of output properties for our fiber laser and previously reported narrow-linewidth fiber lasers based on the PM fiber.

Power	Linewidth	PER	Beam Quality	Power (kW/GHz)	Refs.
0.56 kW	5 GHz	~14 dB	1.25/1.25	0.112	[34]
0.818 kW	7 GHz	~13 dB		0.116	[22]
1.5 kW	13 GHz	>13 dB	1.25/1.23	0.115	[18]
1.89 kW	45 GHz	15.5 dB		0.042	[15]
3.25 kW	20 GHz	~15 dB	1.215/1.185	0.163	[35]
2.01 kW	8 GHz	~15 dB	1.32/1.26	0.25	This work

## Data Availability

Not applicable.
